# Supplementary material for: Migrating immune cells globally coordinate protrusive forces
Source: Nat Immunol. 2025 Jul 15;26(8):1258–66. doi: 10.1038/s41590-025-02211-w (PMC12307229; doi:10.1038/s41590-025-02211-w)
Supplement: Supplementary file 1 — Reporting Summary [file 41590_2025_2211_MOESM1_ESM.pdf]

nature portfolio

Corresponding author(s): Michael Sixt

Last updated by author(s): YYYY-MM-DD

Reporting Summary

Nature Portfolio wishes to improve the reproducibility of the work that we publish. This form provides structure for consistency and transparency in reporting. For further information on Nature Portfolio policies, see our Editorial Policies and the Editorial Policy Checklist.

Statistics

For all statistical analyses, confirm that the following items are present in the figure legend, table legend, main text, or Methods section.

n/a	Confirmed	
☐	☒	The exact sample size (*n*) for each experimental group/condition, given as a discrete number and unit of measurement
☐	☒	A statement on whether measurements were taken from distinct samples or whether the same sample was measured repeatedly
☐	☒	The statistical test(s) used AND whether they are one- or two-sided<br>*Only common tests should be described solely by name; describe more complex techniques in the Methods section.*
☒	☐	A description of all covariates tested
☒	☐	A description of any assumptions or corrections, such as tests of normality and adjustment for multiple comparisons
☐	☒	A full description of the statistical parameters including central tendency (e.g. means) or other basic estimates (e.g. regression coefficient) AND variation (e.g. standard deviation) or associated estimates of uncertainty (e.g. confidence intervals)
☐	☒	For null hypothesis testing, the test statistic (e.g. *F*, *t*, *r*) with confidence intervals, effect sizes, degrees of freedom and *P* value noted<br>*Give P values as exact values whenever suitable.*
☒	☐	For Bayesian analysis, information on the choice of priors and Markov chain Monte Carlo settings
☒	☐	For hierarchical and complex designs, identification of the appropriate level for tests and full reporting of outcomes
☒	☐	Estimates of effect sizes (e.g. Cohen's *d*, Pearson's *r*), indicating how they were calculated

Our web collection on statistics for biologists contains articles on many of the points above.

Software and code

Policy information about availability of computer code

Data collection	Imaging data was collected with: NIS Elements 5.3 (Nikon Instruments - Nikon TiE2); ZEN 3.8 (LSM800 and LSM800 inverted, Zeiss); Nikon JOBS v.5.02 and NIS Elements 5.3 (Nikon CSU-W1); Fusion 2.2 and Imaris v9.91 (Andor Dragonfly 505); VisiView software (Visitron, Zeiss Axio Observer.Z1 inverted); FACS Diva 6.1.3 (FACS Canto BD Biosciences), CytExpert (BC CytoFLEX LX); Cell Sorter Software V2.24 (Sony SH800 SFP sorter).
Data analysis	Fiji (ImageJ) v1.52; Ilastik v1.4; Matlab R2020a; Python v3.9; Davis 8 (Lavision); GraphPad Prism v10.<br>All custom-made scripts used are available upon request.

For manuscripts utilizing custom algorithms or software that are central to the research but not yet described in published literature, software must be made available to editors and reviewers. We strongly encourage code deposition in a community repository (e.g. GitHub). See the Nature Portfolio guidelines for submitting code & software for further information.

Data

Policy information about availability of data

All manuscripts must include a data availability statement. This statement should provide the following information, where applicable:

- Accession codes, unique identifiers, or web links for publicly available datasets
- A description of any restrictions on data availability
- For clinical datasets or third party data, please ensure that the statement adheres to our policy

All data supporting the fidings in this study are available upon request.

Research involving human participants, their data, or biological material

Policy information about studies with human participants or human data. See also policy information about sex, gender (identity/presentation), and sexual orientation and race, ethnicity and racism.

Reporting on sex and gender	*Use the terms sex (biological attribute) and gender (shaped by social and cultural circumstances) carefully in order to avoid confusing both terms. Indicate if findings apply to only one sex or gender; describe whether sex and gender were considered in study design; whether sex and/or gender was determined based on self-reporting or assigned and methods used. Provide in the source data disaggregated sex and gender data, where this information has been collected, and if consent has been obtained for sharing of individual-level data; provide overall numbers in this Reporting Summary. Please state if this information has not been collected. Report sex- and gender-based analyses where performed, justify reasons for lack of sex- and gender-based analysis.*
Reporting on race, ethnicity, or other socially relevant groupings	*Please specify the socially constructed or socially relevant categorization variable(s) used in your manuscript and explain why they were used. Please note that such variables should not be used as proxies for other socially constructed/relevant variables (for example, race or ethnicity should not be used as a proxy for socioeconomic status). Provide clear definitions of the relevant terms used, how they were provided (by the participants/respondents, the researchers, or third parties), and the method(s) used to classify people into the different categories (e.g. self-report, census or administrative data, social media data, etc.) Please provide details about how you controlled for confounding variables in your analyses.*
Population characteristics	*Describe the covariate-relevant population characteristics of the human research participants (e.g. age, genotypic information, past and current diagnosis and treatment categories). If you filled out the behavioural & social sciences study design questions and have nothing to add here, write "See above."*
Recruitment	*Describe how participants were recruited. Outline any potential self-selection bias or other biases that may be present and how these are likely to impact results.*
Ethics oversight	*Identify the organization(s) that approved the study protocol.*

Note that full information on the approval of the study protocol must also be provided in the manuscript.

Field-specific reporting

Please select the one below that is the best fit for your research. If you are not sure, read the appropriate sections before making your selection.

☒ Life sciences ☐ Behavioural & social sciences ☐ Ecological, evolutionary & environmental sciences

For a reference copy of the document with all sections, see nature.com/documents/nr-reporting-summary-flat.pdf

Life sciences study design

All studies must disclose on these points even when the disclosure is negative.

Sample size	No statistical methods were used to predetermine sample sizes. Sample sizes were chosen based on previosu experience and published studies to assess reproducibility. Experiments were repeated multiple times and included parallel imaging of multiple cells, resulting in sample sizes in the range of tens to hundreds. Every migratory cell was analysed.
Data exclusions	To avoid artifacts in cell speed and cell area, only single cell tracks of cells migrating under agarose or microfluidic PDMS devices were included. For speed analysis in PDMS straight channels, only single cells migrating in the channel were considered.
Replication	When possible, analysis was performed with at least twice entirely independent experiments (e.g. independent dendritic cell differentiations, multiple individual cells per individual experiments, and handled at different days). All attempts at replication were successful.
Randomization	Cells were randomized into control and experimental groups.
Blinding	Experiments and data analysis were not blinded. Analysis of subjective data, such as MTOC-nucleus or central actin-nucleus positioning, was

Blinding	performed by two different individuals with reproducible results.

Reporting for specific materials, systems and methods

We require information from authors about some types of materials, experimental systems and methods used in many studies. Here, indicate whether each material, system or method listed is relevant to your study. If you are not sure if a list item applies to your research, read the appropriate section before selecting a response.

Materials & experimental systems

n/a	Involved in the study
☐	☒ Antibodies
☐	☒ Eukaryotic cell lines
☒	☐ Palaeontology and archaeology
☐	☒ Animals and other organisms
☒	☐ Clinical data
☒	☐ Dual use research of concern
☒	☐ Plants

Methods

n/a	Involved in the study
☒	☐ ChIP-seq
☐	☒ Flow cytometry
☒	☐ MRI-based neuroimaging

Antibodies

Antibodies used

Primary antibodies used (Reagent, Species, Clonality, Conjugate, Dilution, Source, Cat. No):

anti-MHC II, rat IgG, Monoclonal M5/114.15.2, eFluor450, 1:400, ebioscience, 48-5321-82;
anti-CD11c, armenian hamster/ IgG, Monoclonal N418, APC, 1:150, ebioscience, 17-0114-82;
anti-CD16/32, rat/IgG2a lambda, Monoclonal clone 93, unconjugated, 1:100, ebioscience,14-0161-85;
anti-gamma-2-tubulin, rabbit IgG, polyclonal, unconjugated, 1:400, abcam, ab11317;
anti-Giantin, rabbit, polyclonal, unconjugated, 1:100, Sysy antibodies, 263003;
anti-LAMP2, rat IgG, monoclonal, unconjugated, 1:100, abcam, ab13524;
anti-Talin, mouse, monoclonal clone 8d4, unconjugated, 1:400, Sigma T3287;
anti-HSPA1A (HSP70), rabbit IgG, polyclonal, unconjugated, 1:10 000, Thermofisher Scientific, PA5-34772.

Secondary antibodies (Reagent, Species, Clonality, Conjugate, Dilution, Source, Cat. No):

anti-rabbit, goat IgG (H+L), polyclonal, Alexa Fluor-488, 1:200-1:400, Invitrogen, A-11008;
anti-rat, donkey IgG (H+L), polyclonal, Alexa FLuor-488, 1:200-1:400, Jackson Immuno Research, AB_2340686;
anti-mouse, goat IgG (H+L), monoclonal, HRP conjugate, 1:10 000, BioRad, 1706516;
anti-rabbit, goat IgG (H+L), monoclonal, HRP conjugate, 1:3 000, BioRad, 1706515.

Other reagents used for staining:
Alexa Flour647 Phalloidin, 1:400, Invitrogen, A22287;
Fluorescein (FITC) Phalloidin, 1:200, Invitrogen, F432.

Validation

All antibodies are commercial standard validated antibodies. Validation data is available on vendor websites. Antibodies were tested using known positive and negative controls and titrated following the manufacturer's recommendations.

Eukaryotic cell lines

Policy information about cell lines and Sex and Gender in Research

Cell line source(s)	Lenti-X-293 derived from HEK 293 cells (TakaraBio); Dendritic cells and T cells were obtained from primary cell cultures as described in the Methods section.
Authentication	Primary cell lines have been tested by antibody markers for differentiation in the respective cell types.
Mycoplasma contamination	All cell lines were tested negative for mycoplasma contamination.
Commonly misidentified lines (See ICLAC register)	No commonly missindentified cell lines were used.

Animals and other research organisms

Policy information about studies involving animals; ARRIVE guidelines recommended for reporting animal research, and Sex and Gender in Research

Laboratory animals	Laboratory mice (mus musculus) from the following backgrounds were used: WT C57BL/6 (Janvier), WASp-/- (B6.129S6-Wastm1Sbs/J; No. 019458; The Jackson Laboratory) and DOCK8-/- a gift from Yoshinori Fukui's lab). All mice used in this study were bred on a C57BL/6 background and maintained at the Institute of Science and Technology Austria (ISTA) institutional animal facility following

the guidelines from its ethics commission and the Austrian law for animal experimentation. Mice with 8 to 12 weeks of age were used for organ removal and cell extraction.

Wild animals: This study did not involve wild animals.

Reporting on sex: Both male and female animals indistinctly used in this study.

Field-collected samples: This study did not involve samples collected from the field.

Ethics oversight: Guidelines from the ethics commission of ISTA's animal facility and the Austrian law for animal experimentation were followed during the course of this study.

Note that full information on the approval of the study protocol must also be provided in the manuscript.

Plants

Seed stocks: *Report on the source of all seed stocks or other plant material used. If applicable, state the seed stock centre and catalogue number. If plant specimens were collected from the field, describe the collection location, date and sampling procedures.*

Novel plant genotypes: *Describe the methods by which all novel plant genotypes were produced. This includes those generated by transgenic approaches, gene editing, chemical/radiation-based mutagenesis and hybridization. For transgenic lines, describe the transformation method, the number of independent lines analyzed and the generation upon which experiments were performed. For gene-edited lines, describe the editor used, the endogenous sequence targeted for editing, the targeting guide RNA sequence (if applicable) and how the editor was applied.*

Authentication: *Describe any authentication procedures for each seed stock used or novel genotype generated. Describe any experiments used to assess the effect of a mutation and, where applicable, how potential secondary effects (e.g. second site T-DNA insertions, mosiacism, off-target gene editing) were examined.*

Flow Cytometry

Plots

Confirm that:

☐ The axis labels state the marker and fluorochrome used (e.g. CD4-FITC).

☐ The axis scales are clearly visible. Include numbers along axes only for bottom left plot of group (a 'group' is an analysis of identical markers).

☐ All plots are contour plots with outliers or pseudocolor plots.

☒ A numerical value for number of cells or percentage (with statistics) is provided.

Methodology

Sample preparation: Cell suspension was filtered to avoid cell aggregates.
For analysis of surface expression markers in DCs, cells were counted and equal numbers of cells were stained in parallel with different antibodies.

Instrument: Data on surface expression markers was acquired either in a FACS Canto BD Biosciences or in a BC CytoFLEX LX machine.
Data on progenitor cell line reporters was acquired in a Sony SH800 FP cell sorter (sorting chip: 100 um).

Software: Analysis of surface expression markers was performed using either FACS Diva 6.1.3 (FACS Canto BD Biosciences) or CytExpert software (BD CytoFLEX LX). Analysis of positive progenitor cell line reporters was performed using Cell Sorter Software V2.24.

Cell population abundance: For establishment of progenitor cell line reporters a minimum of 500 000 positive cells (expressing either GFP or mCherry) were sorted.

Gating strategy: Doublets were excluded based on FSC and SSC profile.
For the establishment of progenitor cell line reporters, cells were discriminated based on the signal distribution in a single fluorescence channel.

☐ Tick this box to confirm that a figure exemplifying the gating strategy is provided in the Supplementary Information.
